# Supplementary material for: Laser light sources for photobiomodulation: The role of power and beam characterization in treatment accuracy and reliability
Source: PLoS One. 2022 Mar 30;17(3):e0266193. doi: 10.1371/journal.pone.0266193 (PMC8967059; doi:10.1371/journal.pone.0266193)
Supplement: Attachment — Submitted filename: Response to Reviewers.docx [file pone.0266193.s001.docx]

Rebuttal letter

February 17th, 2022

Ref. manuscript PONE-D-21-39449 - "Laser light sources for Photobiomodulation: The role of power and beam characterization in treatment accuracy and reliability. "

Academic Editor - Michael R Hamblin

Dear Dr. Hamblin,

Thank you for your time and consideration in the review of our manuscript. We are pleased to attach here a revised version here. We would also like to thank the reviewers for their thoughtful feedback. Each critique has been addressed in this resubmission which is stronger and clearer as a result. (R1-PONE-D-21-39449).

Kind Regards,

Rinaldo Roberto de Jesus Guirro.

**- Journal Requirements**

**Comments:** Please ensure that your manuscript meets PLOS ONE's style requirements, including those for file naming.

**Answer:** The requirement has been met, and the manuscript meets the style of PLOS ONE. Should any points need to be adjusted, please let me know where. Thank you.

**Comments:** Please clarify in your Methods section how permission was sought to access the equipment and who provided permission.

**Answer:** The requirement was followed and inserted in the Methods section. Thank you.

**Comments:** Please review your reference list to ensure that it is complete and correct. If you have cited papers that have been retracted, please include the rationale for doing so in the manuscript text, or remove these references and replace them with relevant current references. Any changes to the reference list should be mentioned in the rebuttal letter that accompanies your revised manuscript. If you need to cite a retracted article, indicate the article's retracted status in the References list and also include a citation and full reference for the retraction notice.

**Answer:** The references have been reviewed, and no article on the list has been retracted, to our knowledge. However, two are not available at PubMed (Wright et al. DOI: 10.1007/BF01588600 and Fukuda - DOI: 10.1590/S1413-35552008000100013). Still, we would like to point out that both can be accessed on the web through their DOI.

**- Reviewer #1**

**Comment:** That the study was limited to devices produced and used in Brazil should be stated in the Abstract, perhaps as follows (second sentence, first paragraph): "The aim of this study was to evaluate the average and maximum power and to describe the beam diameter of different low-power laser photobiomodulation devices in clinical use in Brazil.".

**Answer:** We thank the reviewer. This suggestion has been accepted and highlighted in the manuscript.

**Comment:** Line 203 (pg 4), "The average power, fell within 90% to 110%...": Please delete the comma after "power".

**Answer:** The suggestion was accepted and highlighted in the manuscript. Thank you.

**Comment:** Line 216 (pg 4), "...as well as for 904 nm (Figure 3C) is highlighted.": Please change "is" to ", are".

**Answer:** The suggestion was accepted and highlighted in the manuscript. Thank you.

**Comment:** 2nd para, pg 8, "Here we can calculate the beam radios with the probe in contact...": Please change "radios" to "radius".

**Answer:** The suggestion was accepted and highlighted in the manuscript. Thank you.

**- Reviewer #2**

**Comment:** Page 2 Line 5 - The authors report that "Differences in beam diameter of between 38% and 543% of the nominal values" - COMMENT most laser is used for Photobiomodulation are semiconductor/dialysers which typically have elliptical beams in which case they do not have a diameter (strictly speaking they have two diameters) - SUGGESTION describe the differences in the beam area rather than diameter.

**Answer:** The suggestion was considered. However, we cannot state the difference in the area since the nominal definition in the Brazilian devices is given in general averages. Thus, as our data is presented and made available, only one diameter was measured and compared. So, unfortunately, changing to an area would produce non-existent data. Also, we would like to point out that in the session Methods/ Beam diameter evaluation, we explain how this value was reached. Furthermore, we emphasize that we have inserted this as a limitation of the study. We are open to further considerations.

**Comment:** Page 3 Line 104 - The authors report that "Photobiomodulation parameters associated with its effectiveness are not only fluence and light penetration depth but also full optical properties, such as refractive index, absorption, scattering coefficients, beam diameter, and stable output power." also line 115 "However, the equipment manufacturers have predefined some base fluence values without considering the individual characteristics of each subject". Line 116 this context, if the power and the beam diameter are different than expected due to a faulty function of the equipment, the quality of the treatment will be affected - COMMENT the authors mixed photobiomodulation parameters and topics of light propagation into one sentence light penetration depth refractive index, absorption, scattering coefficients are topics of light propagation. I think it is beyond the scope of this paper tooth tackle matters of individual characteristics of each patient and a stick to the key objective which is "evaluate and describe the laser beam diameter and the average power in different devices in clinical use in Brazil." - SUGGESTION remove references to light propogation in the patient as follows.

**Answer:** The suggestion was accepted, and the sentence had been deleted from the manuscript. Thank you.

**Comment:** Enwemeka reports that trained professionals can only control the physical parameters of the laser to be used for each pathology, given its individuality [...] if the power and the beam diameter are different than expected due to a faulty function of the equipment, the quality of the treatment will be affected. Thus, the objective of this study was to evaluate and describe the laser beam diameter and the average power in different devices in clinical use in Brazil. - I added the word "only" on line 114.

**Answer:** The suggestion was accepted and highlighted in the manuscript. Thank you.

**Comment:** Page 4 Line 139-141 - The authors report that "The devices with wavelengths in the red spectrum were analyzed during one minute of sequential irradiation and the infrared ones during one and three minutes to observe possible emitted power changes" - COMMENT why did you choose longer treatment times for the infrared lasers? - SUGGESTION state the reasons why you choose longer treatment times for the infrared lasers.

**Answer:** The suggestion was discussed, resubmitted, and highlighted in the manuscript. Thank you.

**Comment:** Page 11 Line (no line reference on manuscript) - The authors report that "Their results indicate that as the beam width increases, there is also an increase in the penetration depth. Furthermore, a method in which the beamwidth changes from 1 to 40 mm were proposed by their group, demonstrating that beam diameters between 1 mm and 5 mm have a notable increase in penetration depth" - COMMENT I downloaded the reference paper (Barbosa 2020 Analysis of low-level laser transmission at wavelengths 660, 830 and 904 nm in biological tissue samples. PMID 32516626). The referenced paper does not conclude that as beam width increases, there is also an increase in the penetration depth. It seems both intuativly wrong and my own punpublished experiemnts on pit tissue find the opposite. - SUGGESTION remove all references to increased beam leads to increased penetration.

**Answer:** We agree that the citation was misplaced. The central idea was in front of the wavelength in biological tissues as presented by Barbosa 2020 and the wavelength of the beam according to the extrapolation of Ash 2017. The text was reorganized for the correct interpretation. Thank you for the suggestion.

**Comment:** Page 12 Line (no line reference on manuscript) - The authors report that "the real fluence emitted by the equipment has not been measured" - COMMENT equipment does not emit "fluence", it emits photons at a wavelength, photon per second equal power, when divided by a beam area on the pataint equals irradiance, and when a doctor, nurse or therapist applies that irradiance for a certain amount of time then a certain fluence has been delivered. - SUGGESTION "the real power or irradiance emitted by the equipment has not been measured".

**Answer:** The suggestion was accepted and highlighted in the manuscript. Thank you.

**Comment:** Page 12 CONCLUSION - I agree with this conclusion. The Photobiomodulation literature is plagued by the problems described.

**Answer:** Thank you for your appreciation.
